# The Impact of Different Proportions of a Treated Effluent on the Biotransformation of Selected Micro-Contaminants in River Water Microcosms

**DOI:** 10.3390/ijerph111010390

**Published:** 2014-10-10

**Authors:** Karsten Nödler, Maria Tsakiri, Tobias Licha

**Affiliations:** Department Applied Geology, Geoscience Centre of the University of Göttingen, Goldschmidtstr. 3, 37077 Göttingen, Germany; E-Mails: maria.tsakiri@stud.uni-goettingen.de (M.T.); tobias.licha@geo.uni-goettingen.de (T.L.)

**Keywords:** biodegradation, pharmaceuticals, caffeine, river water, treated effluent, valsartan acid

## Abstract

Attenuation of micro-contaminants is a very complex field in environmental science and evidence suggests that biodegradation rates of micro-contaminants in the aqueous environment depend on the water matrix. The focus of the study presented here is the systematic comparison of biotransformation rates of caffeine, carbamazepine, metoprolol, paracetamol and valsartan in river water microcosms spiked with different proportions of treated effluent (0%, 0.1%, 1%, and 10%). Biotransformation was identified as the dominating attenuation process by the evolution of biotransformation products such as atenolol acid and valsartan acid. Significantly decreasing biotransformation rates of metoprolol were observed at treated effluent proportions ≥0.1% whereas significantly increasing biotransformation rates of caffeine and valsartan were observed in the presence of 10% treated effluent. Potential reasons for the observations are discussed and the addition of adapted microorganisms via the treated effluent was suggested as the most probable reason. The impact of additional phosphorus on the biodegradation rates was tested and the experiments revealed that phosphorus-limitation was not responsible.

## 1. Introduction

Wastewater is a significant source of micro-contaminants such as pharmaceuticals and caffeine [1]. Consequently, these compounds are frequently detected in effluent-receiving surface waters (e.g., [2,3]) and their occurrence and fate is subject of current research and scientific discussion. Biotransformation, photodegradation, and sorption were identified as significant mechanisms for the attenuation of micro-contaminants in surface waters [4,5,6,7]. Biotransformation can only occur under favorable conditions [8] and the presence of already adapted microorganisms is advantageous [9]. From the results of aerobic laboratory degradation experiments with the pharmaceuticals gemfibrozil and naproxen in river water microcosms (WWTP-influenced river), Grenni *et al.* [10] concluded that the observed degradation capability of the microbial community was presumably acquired by chronic exposure to the investigated compounds. They also observed significant changes of the microbial community induced by pharmaceutical residues. In a recent study focusing on sample stabilization, a significantly lower half-life of the readily biodegradable compound caffeine was observed when exposed to treated wastewater matrix instead of river water matrix [11]. However, also the contrary was observed, as some compounds such as the antihypertensive metoprolol were exceptionally stable in treated wastewater matrix but eliminated in the river water [11]. Matrix-dependent stabilities of readily degradable compounds were also observed during sample stability tests presented by Gawlik *et al.* [12].

Co-metabolism—defined as the transformation of a non-growth substrate in the obligate presence of a growth substrate or another transformable compound [13]—can result in significantly higher transformation rates of pharmaceutical residues [9,14]. On the other hand, the presence of readily degradable matrix components can also have a negative impact on the biotransformation of selected micro-contaminants [14]. Elevated bacterial toxicity in treated effluents is likewise conceivable to inhibit microbial growth and thus the attenuation of selected compounds. At the end of their degradation experiments Grenni *et al.* [10] observed a collapse in live bacteria and they suggested the presence of toxic transformation products (TPs) as a possible reason.

The focus of the study presented here is the systematic comparison of biotransformation rates of selected micro-contaminants in river water microcosms spiked with different proportions of treated effluent (0%, 0.1%, 1%, and 10%). The key question is how much treated effluent is necessary to significantly induce the effects observed by Hillebrand *et al.* [11]. Therefore, river water and treated effluent were collected at the same sampling locations where they observed the water matrix-dependent stability of selected micro-contaminants. Furthermore, the impact of an elevated phosphorus (P) concentration on the biodegradation rates was tested. The chosen model compounds demonstrate high detection frequencies in wastewater treatment plants (WWTPs) and in the environment and their fate in WWTP and surface waters is known: The stimulant caffeine and the analgesic paracetamol are readily biodegradable compounds, the antihypertensives metoprolol and valsartan demonstrate moderate to high stability, and the anticonvulsant carbamazepine is considered as highly persistent [15,16,17,18]. The concentrations of the spiked compounds and TPs (atenolol acid from metoprolol; mono- and dimethylxanthines from caffeine; valsartan acid from valsartan) were monitored for the duration of 32 days.

## 2. Experimental Section

### 2.1. Materials

Methanol and acetonitrile (both LC/MS grade) were purchased from Fisher Scientific (Schwerte, Germany). Ammonium acetate was obtained from VWR (Darmstadt, Germany). 1-Methylxanthine, 3-methylxanthine, 7-methylxanthine, atenolol-D_7_, caffeine, caffeine-D_9_, carbamazepine, metoprolol, metoprolol-D_7_, 1,7-dimethylxanthine (paraxanthine), paraxanthine-D_6_, 1,3-dimethylxanthine (theophylline), 3,7-dimethylxanthine (theobromine), and theobromine-D_6_ were purchased from Sigma-Aldrich (Steinheim, Germany). Irbesartan, losartan, and valsartan were purchased from TCI (Eschborn, Germany). Atenolol acid, paracetamol-D_4_, losartan-D_4_, irbesartan-D_7_, valsartan-D_9_, and carbamazepine-D_10_ were from LGC Promochem (Wesel, Germany), and atenolol and paracetamol were purchased from Fagron (Barsbüttel, Germany). The synthesis and purification of valsartan acid is described in Nödler *et al.* [19]. 

**Table 1:** Structures and selected physicochemical properties (taken from SciFinder^®^) of the spiked compounds.

Name (Application)	Structure	Molecular Weight	Log *K_OW_*	p*Ka*
Caffeine (stimulant)	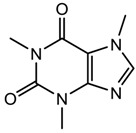	194.19	−0.628 ± 0.753	0.52 ± 0.70 ^a^
Carbamazepine (anticonvulsant)	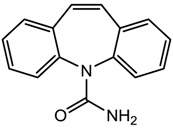	236.27	1.895 ± 0.597	−0.49 ± 0.20 ^a^ 13.94 ± 0.20 ^b^
Metoprolol (antihypertensive)	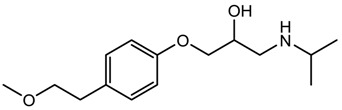	267.36	1.632 ± 0.263	9.43 ± 0.10 ^a^ 13.89 ± 0.20 ^b^
Paracetamol (analgesic)	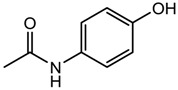	151.16	0.475 ± 0.210	1.72 ± 0.50 ^a^ 9.86 ± 0.13 ^b^
Valsartan (antihypertensive)	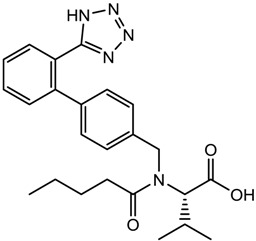	435.52	4.022 ± 0.606	0.60 ± 0.10 ^a^ 3.56 ± 0.10 ^b^

^a^ mostly basic p*Ka* (25 °C); ^b^ mostly acidic p*Ka* (25 °C).

Structures and selected physicochemical properties of the spiked compounds are presented in Table 1. An internal standard (IS) mix containing 10 ng·μL^−1^ caffeine-D_9_, carbamazepine-D_10_, ibuprofen-D_3_, metoprolol-D_7_, paracetamol-D_4_, paraxanthine-D_6_, theobromine-D_6_, and valsartan-D_9_ was prepared in acetonitrile. All materials used for sampling and lab experiments were compatible with the studied micro-contaminants [20].

Gravel (4–6 mm grain size) was obtained from RuT (Lehrte, Germany). The material was further purified by wet sieving until no turbidity could be mobilized. The fraction >4 mm was sterilized by heat for 12 h at 130 °C (drying cabinet) and used for the batch tests.

### 2.2. Sampling and Pretreatment of Matrix Components

River water (RW) and treated effluent (TE) were collected from the same locations, in which the matrix-dependent stability of micro-contaminants was observed in the preliminary study [11] (RW sampling location: River Leine, 51°30′38.7″N 9°55′23.9″E; TE: WWTP Göttingen, Lower Saxony, Germany; sampling date of both matrices: 29 May 2013). A dip sampler was used for sampling and glass bottles (2-L, screw cap) were used as sample containers. In total ~8–9 L of RW and ~2 L of TW were collected.

In the course of a previously published study the treated effluent was analyzed on a daily basis for 27 days and easily degradable compounds such as ibuprofen, caffeine, and caffeine metabolites were not detected [21]. Therefore, the presence of favorable degradation conditions and adapted microorganisms can be assumed. Depending on weather conditions, the WWTP treats 30,000–80,000 m^3^ wastewater per day. The treatment process consists of a mechanical treatment for the separation of solid material (*i.e.*, a grit, fat separator, and a primary settler) followed by activated sludge treatment, including nitrification and denitrification. Additionally, chemically facilitated phosphorus removal is performed. Under dry weather discharge conditions, the mean hydraulic residence time is approximately 20–24 h.

Coarse particles in the RW were allowed to settle for 30 min and floating plant material was removed. The supernatant was equally distributed into two 5-L beakers and the TE-sample (without any particle removal) to a 2-L beaker, all equipped with magnetic stirrers. The beakers were covered with aluminum foil and stirred for 20 h at room temperature to achieve equal concentrations of dissolved O_2_ in both matrices. Prior to the preparation of the batch tests dissolved O_2_, pH value, and the electrical conductivity of RW and TE were determined electrochemically. The results were 8.5/8.4 mg·L^−1^ dissolved O_2_, 8.64/8.56 pH, and 710/1137 μS·cm^−1^ for RW and TE, respectively.

### 2.3. Preparation and Sampling of Batch Experiments

Individual stock solutions of the tested compounds were prepared in methanol (caffeine, paracetamol, metoprolol, and carbamazepine) and acetonitrile (valsartan) and a combined spike solution with 100 μg·mL^−1^ of each analyte was prepared in acetonitrile. For the preparation of the batch tests several glass bottles (1-L) were rinsed with ultrapure water and acetone and sterilized at 130 °C in a drying cabinet. After cooling to room temperature, each bottle was spiked with 500 μL of the spike solution. The solvent was evaporated to remove any carbon source additionally to the micro-contaminants. All experiments were performed in duplicate.

#### 2.3.1. Main Experiment

Experimental degradation matrices (500 mL) were prepared by mixing RW with different fractions of TE in 500-mL volumetric flasks: 0%, 0.1%, 1%, and 10% (v/v). The experimental matrices were transferred to the spiked glass bottles and the micro-contaminants were allowed to dissolve for 30 min at room temperature. Consequently, an individual spike concentration of 100 μg·L^−1^ of each analyte was applied. To distinguish abiotic effects such as chemical decomposition and sorption from biodegradation, poisoned controls of the 0% and the 10% fractions were prepared by adding 5 g·L^−1^ NaN_3_ to the respective bottles. Degradation of paracetamol, caffeine, metoprolol, and carbamazepine in RW matrix was found successfully inhibited by using the applied NaN_3_ concentration [11]. Samples (5 mL) were collected from all bottles as the reference concentration (c_0_) and kept frozen (−18 °C) until analysis. To all batches 50 g of gravel were added as natural substratum to stimulate biofilm formation and all batches were shaken thoroughly. The gravel was evenly distributed to the bottom and all batches were incubated at 17 °C on an orbital shaker (150 rpm) in the dark to prevent photochemical reactions. In all batches a head space of ≥500 mL (normal room air) remained as O_2_-reservoir. The shaking speed was selected to homogenize the water phase and thus preventing an O_2_-gradient within the water phase without disturbing the gravel layer. The batches were sampled (5-mL aliquots, sterilized 10-mL Eppendorf pipettes) after 3, 6, 10, 14, 21, and 32 days and the samples were stored at −18 °C until analysis.

#### 2.3.2. Additional Experiment

The impact of the P concentration on the primary degradation rate of selected micro-contaminants was evaluated by preparing RW matrix batches with elevated ortho-phosphate (PO_4_^−3^) concentration. The PO_4_^−3^ background concentration of the RW was 100 μg·L^−1^. Four batches with RW were prepared similar to the main experiment but to two of the four batches 500 μL of a 100 mg·L^−1^ aqueous phosphate solution (as KH_2_PO_4_) were added, which led to a doubling of the native PO_4_^−3^ concentration. No change of the pH value was observed.

### 2.4. Analysis

For analysis of the batch experiments 1 mL of the thawed sample was mixed with 10 μL IS and 5 μL of 1 mM ammonium acetate solution in a 2-mL autosampler vial and centrifuged for 10 min at 4000 rpm. The analytes were separated and quantified by HPLC/ESI-MS-MS. The HPLC system consisted of a Varian ProStar 410 autosampler and a high-pressure gradient system of two pumps (Varian ProStar 210). For chromatographic separation the Polaris C18-Ether column 150 × 2 mm i.d., 3 μm particle size (Varian, Darmstadt, Germany) was used. 

**Table 2:** ESI-MS-MS-parameters of parent compounds (PC) and transformation products (TP).

Compound	Type	Quantifier	Cap U [V] ^a^	CE [V] ^b^	Qualifier	Cap U [V] ^a^	CE [V] ^b^	IS ^c^
Atenolol	PC	267 > 145	55	−20.0	267 > 190	55	−11.0	1
Atenolol acid	Atenolol/Metoprolol TP	268 > 191	60	−12.0	268 > 145	60	−17.5	1
Metoprolol	PC	268 > 116	55	−11.0	268 > 191	55	−10.0	2
1-Methylxanthine	Caffeine TP	165 > 108	−55	19.0	165 > 80	−55	25.0	3
3-Methylxanthine	Caffeine TP	165 > 122	−55	19.0	165 > 150	−55	18.0	3
7-Methylxanthine	Caffeine TP	167 > 124	55	−13.0	167 > 150	55	−12.0	3
Caffeine	PC	195 > 138	55	−9.5	195 > 110	55	−9.0	4
Paraxanthine	Caffeine TP	181 > 124	60	−8.0	181 > 96	60	−10.5	5
Theobromine	Caffeine TP	181 > 138	55	−9.5	181 > 110	55	−13.0	3
Theophylline	Caffeine TP	181 > 124	60	−8.0	181 > 96	60	−10.5	5
Paracetamol	PC	152 > 110	40	−11.0	152 > 93	40	−18.5	6
Carbamazepine	PC	237 > 194	45	−11.0	237 > 179	45	−27.0	7
Irbesartan	PC	429 > 207	50	−22.5	429 > 195	50	−21.5	8
Losartan	PC	423 > 207	40	−21.0	423 > 180	40	−36.0	9
Valsartan	PC	434 > 179	−60	23.0	434 > 350	−60	19.0	10
Valsartan acid	Sartan TP	265 > 165	−40	17.0	265 > 193	−40	13.5	10
**No. ^c^**	**Internal standards**							
1	Atenolol-D_7_	274 > 145	55	−17.5				
2	Metoprolol-D_7_	275 > 123	55	−11.5				
3	Theobromine-D_6_	187 > 144	55	−13.5				
4	Caffeine-D_9_	204 > 144	60	−8.5				
5	Paraxanthine-D_6_	187 > 127	60	−9.0				
6	Paracetamol-D_4_	156 > 114	40	−11.0				
7	Carbamazepine-D_10_	247 > 204	45	−13.0				
8	Irbesartan-D_7_	436 > 207	50	−22.5				
9	Losartan-D_4_	427 > 211	40	−21.0				
10	Valsartan-D_9_	443 > 179	−60	22.5				

^a^ capillary voltage; ^b^ collision energy; ^c^ assigned internal standard according to the IS numbering.

The flow was 200 μL·min^−1^. The separation was operated at 30 °C and the injection volume was set to 100 μL. Eluent A was 0.015% formic acid +5% methanol in ultrapure water, eluent B was methanol. The elution started isocratically for 50 s with 100% A, followed by a gradient of 10 s to 95% A. This step was followed by a 24-min linear gradient to 95% B. This was held for 4 min followed by a 1-min linear gradient to 100% A, which was maintained for 10 min to equilibrate the system. A Varian 1200 L triple quadrupole with electrospray interface (ESI) was used for detection and quantification. A spray voltage of 5.5 kV in positive mode, −4.5 kV in negative mode and shield voltages of 0.5 kV and −0.5 kV were used, respectively. Detection was performed in multiple reaction monitoring (MRM). The MS-MS-parameters of all analytes and their assigned internal standards are presented in Table 2.

Dwell time of the quantifiers and qualifiers were 50 ms and 15 ms, respectively. Isotopically labeled internal standards were chosen as a powerful tool for matrix compensation [22]. Compounds without isotopically labeled analogues were quantified by using the closest-matching IS according to retention time, ionization mode (±ESI), and structure. For the calibration the concentration levels (5–125 μg·L^−1^ for the mono-methylxanthines and 1–125 μg·L^−1^ for all other compounds) were prepared in ultrapure water and processed according to the samples (addition of IS and ammonium acetate) and 1 μg·L^−1^ (1% of the initial concentration) was defined as the reporting limit. The linear correlation coefficients were >0.99 for all analytes and the recovery of all spiked compounds in the c_0_ (RW/TE matrix) were 90%–102%.

### 2.5. Background Concentrations of Relevant Parameters (Main Experiment)

Background concentrations of the selected micro-contaminants and TPs in RW and TE were determined by solid phase extraction (SPE) and HPLC/ESI-MS-MS. The SPE was performed according to Nödler *et al.* [19]. To ensure sufficient stabilization of the extracted analytes the SPE cartridges were stored at −18 °C until analysis [11]. Irbesartan, losartan, atenolol, and their respective IS were exclusively included as analytes for the background determination. Irbesartan and losartan were of particular interest as they are, together with valsartan, frequently used in Germany and may be potential precursors of valsartan acid [19]. Atenolol was of particular interest as it is a precursor of atenolol acid [23,24]. Furthermore, the concentrations of additional micro-contaminants (selected antibiotics, antiallergics, corrosion inhibitors, *etc.*) were analyzed according to Nödler *et al.* [25]. Acesulfame was analyzed according to Nödler *et al.* [19] to estimate the initial wastewater burden of the RW [26]. The total organic carbon (TOC) concentration was determined as non-purgeable organic carbon (NPOC) by using the combustion method. Elemental analysis was conducted by ICP-MS and ortho-phosphate was photometrically determined [27]. Acute toxicity of the TE was determined by the luminescent bacteria test [28].

## 3. Results and Discussion

### 3.1. Background Concentrations

The TOC of RW and TE were 12 and 9.6 mg·L^−1^, respectively. Regarding micro-contaminants significant to the experiments, the highest individual background concentration was 1.7 μg·L^−1^ (valsartan acid) in the TE. Therefore, the background concentrations of all investigated and related compounds were <0.2 μg·L^−1^ in all microcosm matrices (0%–10% TE) and thus significantly below the here defined reporting limit of 1 μg·L^−1^. No acute toxicity was detected in the TE. At the sampling location of the RW 0.1 μg·L^−1^ acesulfame were found. From the results typically encountered in German WWTP effluents [29] the wastewater burden was estimated ~0.5%.

### 3.2. pH and O_2_ Measurement in the Microcosms

The pH values and O_2_-concentrations were determined at beginning and end of the experiment. The pH values were in the range of 8.5–8.6 at the beginning and slightly decreased to 8.3 in the non-poisoned microcosms. The same applies to the O_2_-concentrations (8.1–8.2 mg·L^−1^ at the beginning and 7.9–8.0 mg·L^−1^ at the end of the experiment).

### 3.3. Abiotic Microcosms

To distinguish abiotic from biological effects, control batches poisoned with NaN_3_ were prepared. The poisoning was considered successful as no significant concentration changes of the spiked compounds were observed between day 3 and 32. Furthermore, no TPs were detected in any of these microcosms. However, low (6%–10%) abiotic losses of caffeine, metoprolol, and paracetamol were observed by comparing the initial concentrations (without gravel) with the concentrations of the first sampling time (3 days, with gravel). The highest abiotic loss was accounted for metoprolol. At experimental pH metoprolol predominantly exists in its cationic species [30]. In a previously published study cationic exchange was identified as significant sorption process of beta-blockers [31], which is a possible explanation for the observed loss.

### 3.4. Biotic Microcosms

**Figure 1:**
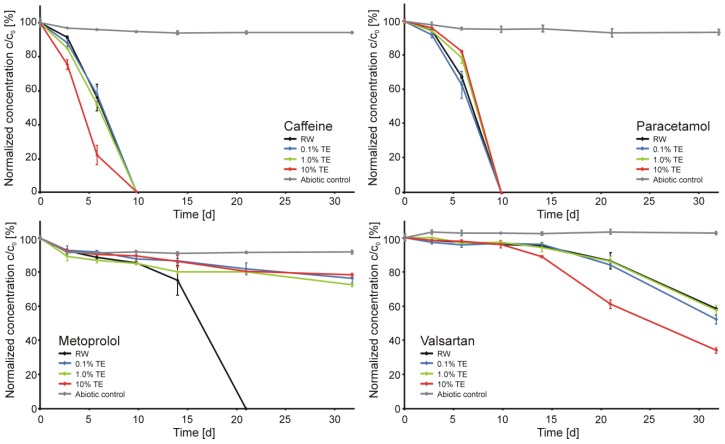
Concentrations of caffeine, paracetamol, metoprolol, and valsartan in the microcosms (RW: river water, TE: treated effluent). Mean values of two batches per time point, error bars represent the respective concentration range of duplicates.

**Figure 2:**
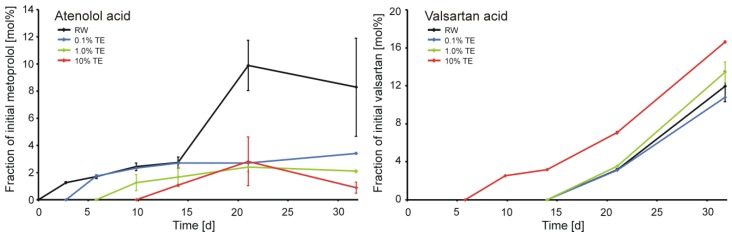
Concentrations of atenolol acid and valsartan acid in the microcosms (RW: river water, TE: treated effluent). Mean values of two batches per time point, error bars represent the respective concentration range of duplicates.

As expected, no significant degradation of carbamazepine was observed in any of the microcosms (Figure A1 in the Supplementary Materials). The concentrations of caffeine, paracetamol, metoprolol, and valsartan during the main experiment are presented in Figure 1. The concentrations of atenolol acid and valsartan acid are presented in Figure 2. As expected from the previous study [11], the biodegradation rate of caffeine increased with increasing concentration of TE in the matrix. Despite the clear evidence for biodegradation, the analyzed caffeine TPs (mono- and dimethylxanthines) were not detected in any of the microcosms. This observation is in agreement with findings of [32] who observed significant attenuation of caffeine in a karst aquifer but failed to detect mono- and dimethylxanthines. In addition to dilution effects they suggested fast degradation of the TPs itself or different degradation pathways as possible reasons. Valsartan was also much faster degraded in presence of 10% TE compared with the RW and smaller proportions of TE and the evolution of the valsartan TP valsartan acid followed this trend (Figure 2). However, in contrast to caffeine and valsartan, the degradation rates of paracetamol and metoprolol decreased in the presence of TE. For paracetamol significant differences were observed at 1% and 10% TE compared to the pure RW. However, due to the sampling schedule the difference was only detected at one sampling time and, therefore, this observation should not be overestimated. The differences of metoprolol biodegradation were much clearer: No metoprolol was present in the RW microcosms after 21 days and the highest concentration of atenolol acid was observed. However, the biodegradation was strongly inhibited at presence of ≥0.1% TE. The impact of the lowest proportion was surprising, as the river water already contained wastewater at the specific sampling location. Similar observations regarding metoprolol degradation were reported by Hillebrand *et al.* [11]. However, in their study paracetamol demonstrated a faster degradation in pure TE than in RW.

An elevated total phosphorus (P) concentration was detected in the TE (384 μg·L^−1^ *vs.* 67.1 μg·L^−1^ in the RW) and, consequently, a significantly higher P concentration was present in the 10% TE experimental matrix. The same applies to micro-nutrients such as zinc (Zn) and molybdenum (Mo) (see Table A1 in the Supplementary Materials). It is well known that P plays a key role in the biodegradation of environmental contaminants (e.g., [33]), but no significant effects were observed during the additional experiment with native RW and RW spiked with additional PO_4_^−3^ (increase to twofold concentration value) (see Figure A2 in the Supplementary Materials). The impact of elevated Zn and Mo concentrations was not tested.

The presence of readily degradable matrix components can result in significantly higher transformation rates of pharmaceuticals residues (co-metabolism) but can also decrease the biotransformation rate of other compounds [14]. From a quantitative point of view this issue is unlikely a reason for the here presented observations, as the TOC concentration of both RW and TE were in the same order of magnitude. Especially for the effects on metoprolol at the very low proportion of 0.1% TE it is very unlikely that co-metabolic processes were responsible. Elevated co-metabolism at 10% TE and thus higher degradation rates of caffeine and valsartan may occur, if a suitable substrate was added via the TE. However, the major task of WWTPs is the removal of the organic load from the wastewater, which is expressed by a decreased biochemical oxygen demand (BOD) of the TE [34]. Therefore, the TOC of treated effluents usually consists of compounds persistent to biodegradation and thus, higher degradation rates due to co-metabolisms seem unlikely. WWTP final effluents are well-known point-sources of bacteria [35]. The observed increasing degradation rates and especially the faster onset of biodegradation of caffeine and valsartan at increasing TE-proportions (Figure 1) may have been induced by the addition of adapted microorganisms via the TE. Furthermore, the inoculated bacteria may have suppressed the autochthonous RW microorganisms, which were responsible for the degradation of paracetamol and especially metoprolol.

Other specific and chemically induced supporting/inhibiting mechanisms are also conceivable. Soluble microbial products (SMPs; humic and fulvic acids, polysaccharides, proteins *etc.*), which are generated during wastewater treatment processes, can inhibit microbial growth [36]. No acute toxicity was observed in the TE by the luminescent bacteria test. However, the SMPs demonstrate also metal chelating properties, playing probably a role in metal bioavailability, as they hinder the consumption of metal micro-nutrients by microorganisms [36]. Furthermore, the bioavailability of the substrate also may be influenced by humic and/or surface-active substances [37]. Further micro-contaminants were determined in the RW and TE as described in the literature [25]. The concentrations of the analyzed compounds did not exceed the spike levels applied in the stabilization study conducted by Hillebrand *et al.* [11]. Therefore, these compounds were not responsible for the observed effects.

### 3.5. Environmental Relevance of the Study

The direct transferability of results from laboratory to field scale is often not possible. However, laboratory studies are necessary, as they allow for decreasing the complexity typically encountered in real ecosystems. The here applied initial concentration of micro-contaminants (100 μg·L^−1^) was higher than typically encountered in surface waters [38]. This concentration was chosen to achieve the analytical sensitivity for the here applied reporting limit (1 μg·L^−1^; 1% of the initial concentration) without any pre-concentration step such as the SPE. Biodegradation rates of micro-contaminants can be concentration-dependent [39]. However, as the half-lives of caffeine and paracetamol were in the same range as encountered in the preliminary experiments with lower initial concentrations (1 μg·L^−1^) [11], the here applied approach is justified. The here presented test design purposely neglected the impact of natural river sediments and sterilized gravel was offered solely as a substratum and not as a natural reservoir of microorganisms. Radke and Maier [40] followed the opposite approach. They collected sediments from different rivers and locations within a river and used artificial river water for their experiments on the biotransformation of nine different compounds in water/sediment tests. They observed significant differences in biodegradation rates depending on the sampling location of the sediment. Similar to the here presented results Radke and Maier [40] also encountered rather unsystematic results for the biodegradation rates of their compounds, e.g., one sediment was very effective in bezafibrate biodegradation while it was rather ineffective regarding ibuprofen and *vice versa* for another investigated sediment. Furthermore, it is noticeable that sediment from river A was more efficient in removing micro-contaminants than sediment from river B, while the opposite pattern was observed in previous field studies at the investigated rivers. They concluded that physical boundary conditions are more important than the presence of specific microbial communities in the sediment. However, the here presented laboratory results demonstrate that even slight changes of the water phase by TE alone can result in substantial changes of biotransformation rates. The extent to which the here demonstrated impact is noticeable in water/sediment tests and real ecosystems as well as the identification of triggering factors for the observations needs to be addressed in future studies.

## 4. Conclusions

Biotransformation of micro-contaminants is a very complex field in environmental science and it is difficult to understand the subject and all essential factors in its entirety. The here presented results clearly demonstrated changing biodegradation rates in RW-microcosms induced even by very low proportions of TE. There are multiple potential reasons for the observations such as the presence of adapted microorganisms, micro-nutrients, compounds which may influence bioavailability of substrates or co-factors, *etc.* The experiment with additional phosphorus demonstrated that P-limitation was not responsible. There is a substantial need for further research to understand the triggering processes for the observed results.

## Figures and Tables

**Figure A1:**
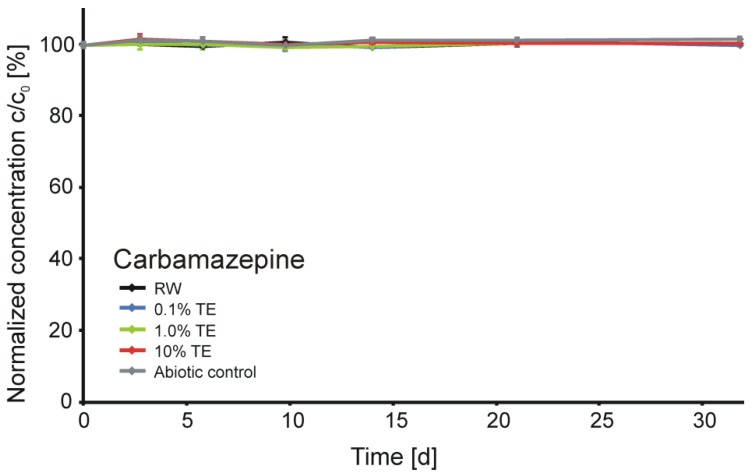
Concentrations of carbamazepine in the microcosms (RW: river water, TE: treated effluent). Mean values of two batches per time point, error bars represent the respective concentration range of duplicates.

**Figure A2:**
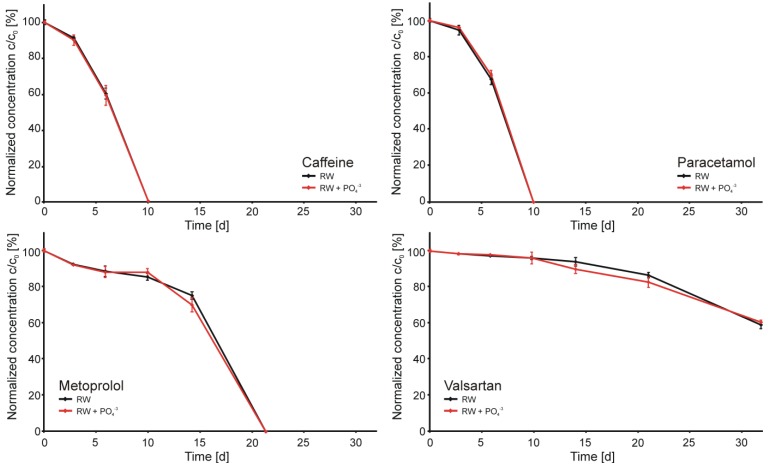
Concentrations of caffeine, paracetamol, metoprolol, and valsartan in the RW microcosms with and without additional phosphate. Mean values of two batches per time point, error bars represent the respective concentration range of duplicates.

## Appendix

**Table A1:** ICP-MS analysis of the native river water (RW) and treated effluent (TE) in μg·L^−1^.

Element	TE	RW	Element	TE	RW
Li7	8.09	4.70	Rb85	11.20	1.04
B11	108	45.20	Sr88	704.00	526.00
Na23	89,400	19,600	Cs133	0.06	0.001
Mg25	11,400	9,650	Ba137	9.10	21.60
Al27	35.10	5.04	La139	0.003	0.006
P31	384.00	67.10	Ce140	0.005	0.007
K39	21,100	4530	Pr141	0.001	0.001
Ca43	21,100	21,000	Nd143	0.006	0.007
Sc45	0.31	0.27	Sm147	<0.001	0.001
Ti49	2.46	1.24	Eu151	<0.005	<0.005
V51	<20	<20	Gd157	0.424	0.004
Cr53	<20	<20	Tb159	<0.001	<0.001
Mn55	8.69	9.49	Dy163	0.001	0.002
Fe57	179.00	177.00	Ho165	<0.001	<0.001
Co59	0.26	0.15	Er166	0.001	0.001
Ni60	2.06	1.31	Tm169	<0.001	<0.001
Cu63	1.94	1.19	Yb174	0.003	0.001
Zn66	5.56	0.82	Lu175	<0.001	<0.001
As75	<2	<2	Hf178	0.001	0.003
Se77	<5	<5	Ta181	<0.006	<0.006
Y89	0.01	0.01	W184	0.162	0.044
Zr90	0.05	0.19	Tl205	0.001	0.001
Nb93	0.01	0.02	Pb208	<2	<2
Mo98	4.47	0.39	Bi209	<0.002	<0.002
Cd114	<0.001	<0.001	Th232	0.001	0.002
Sb121	0.37	0.12	U238	0.75	0.59
